# Differential co-expression framework to quantify goodness of biclusters and compare biclustering algorithms

**DOI:** 10.1186/1748-7188-5-23

**Published:** 2010-05-28

**Authors:** Burton Kuan Hui Chia, R Krishna Murthy Karuturi

**Affiliations:** 1 School of Computing, National University of Singapore, Singapore; 2 Computational & Systems Biology, Genome Institute of Singapore, A-STAR, 60 Biopolis ST, Singapore; 3 CKBH was with Genome Institute of Singapore during this work

## Abstract

**Background:**

Biclustering is an important analysis procedure to understand the biological mechanisms from microarray gene expression data. Several algorithms have been proposed to identify biclusters, but very little effort was made to compare the performance of different algorithms on real datasets and combine the resultant biclusters into one unified ranking.

**Results:**

In this paper we propose differential co-expression framework and a differential co-expression scoring function to objectively quantify quality or goodness of a bicluster of genes based on the observation that genes in a bicluster are co-expressed in the conditions belonged to the bicluster and not co-expressed in the other conditions. Furthermore, we propose a scoring function to stratify biclusters into three types of co-expression. We used the proposed scoring functions to understand the performance and behavior of the four well established biclustering algorithms on six real datasets from different domains by combining their output into one unified ranking.

**Conclusions:**

Differential co-expression framework is useful to provide quantitative and objective assessment of the goodness of biclusters of co-expressed genes and performance of biclustering algorithms in identifying co-expression biclusters. It also helps to combine the biclusters output by different algorithms into one unified ranking i.e. meta-biclustering.

## Background

The inception of microarrays has facilitated quantification of expression of genes at genomic scale in large sets of conditions in time and cost effective manner resulting in a wealth of massive gene expression datasets. Appropriate analysis of these datasets lead to the understanding of the roles of various genes and pathways at genomic-scale.

**Figure 1:**
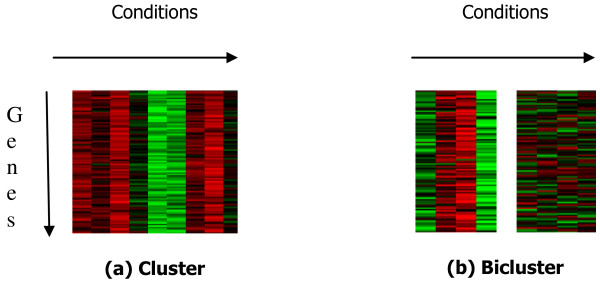
**Illustrating difference between clustering and biclustering**. Heatmaps (red for induction and green for repression) illustrating difference between clustering and biclustering (a) a cluster of genes, genes are co-expressed across most conditions; (b) a bicluster of genes, genes are co-expressed only on a subset of conditions (heatmap on the left) and the heatmap on the right shows no co-expression on the remaining conditions.

Significant portion of microarray data analysis is unsupervised in which the genes are grouped according to the similarity of their expression patterns among multiple conditions. It is based on the observation that the genes involved in similar biological regulatory pathways or functions exhibit similar expression patterns i.e. a cluster of genes may demonstrate a consistent co-expression pattern among most conditions. Several techniques such as agglomerative or divisive clustering algorithms [1-4] that partition the genes into mutually exclusive groups or hierarchies have been reported. On the other hand, unlike the above traditional clustering which uses all available conditions to cluster genes, biclustering has been introduced by Cheng and Church [5] to identify clusters of genes defined based on the respective subsets of conditions. The conditions used for a bicluster of genes are often specific to it i.e. a bicluster of genes is co-expressed in a small subset of conditions and they are expected to show no or weak co-expression in the remaining conditions. The difference between clustering and biclustering is illustrated using the heatmaps in the Figure 1: a cluster of genes are co-expressed over all conditions (figure 1a); but, a bicluster of genes are co-expressed only over a subset of conditions (left heat map in figure 1b) and they are either weekly or not co-expressed among the remaining conditions (right heat map in figure 1b).

Biclustering plays an important role in microarray gene expression analysis. Expression of a cluster of genes may be modulated only in a small subset of conditions demonstrating interesting biology of the condition dependent transcriptional co-regulation and potentially leading to understanding of the underlying mechanisms. For example, in knock out studies, certain groups of genes are activated or suppressed only in a small subset of knock-out conditions. Similarly, in cancer studies, due to heterogeneity of the tumors, certain groups of genes involving in a certain pathway may be co-expressed only in a subset of tumors. In the traditional clustering, the genes co-expressed over all conditions dominate the clustering analysis and the genes co-expressed only in a small subset of conditions may not be elicited.

As the subsets used for different biclusters of genes are not known beforehand, several biclustering algorithms have been proposed in the bioinformatics literature to identify them [5-13]. Different algorithms use different objective functions to identify biclusters of co-expressed genes which makes objective and direct comparison of biclusters and the biclustering algorithms difficult on real data as it lacks a gold standard for evaluation. For example *Cheng and Church's algorithm *(*CC*) [5] minimizes mean squared error in linear model fit. *Iterative Signature Algorithm (ISA) *[7] finds biclusters by maximizing z-scores of expression. *Order Preserving Sub Matrix (OPSM) *[8] elicits biclusters by finding order preserving co-expression submatrices with highest statistical significance support. *Statistical Algorithmic Method for Bicluster Analysis (SAMBA) *[12] is based on finding heavy subgraphs in the gene-condition bipartite graph. The algorithms are summarized in Table 1 for a quick reference.

**Table 1:** Biclustering algorithms

S. No.	Algorithm	Acronym	Reference
1	*Cheng and Church's algorithm*	CC	Cheng and Church [5]

2	*Iterative Signature Algorithm*	ISA	Ihmels et al [7]

3	*Order Preserving Sub Matrix*	OPSM	Ben-Dor et al [8]

4	*Statistical Algorithmic Method for Bicluster Analysis*	SAMBA	Tanay et al [12]

The four biclustering algorithms evaluated using our differential co-expression scoring framework. Their acronyms and references are also given. All four algorithms aim to find biclusters of genes with co-expression in a subset of conditions.

**Figure 2:**
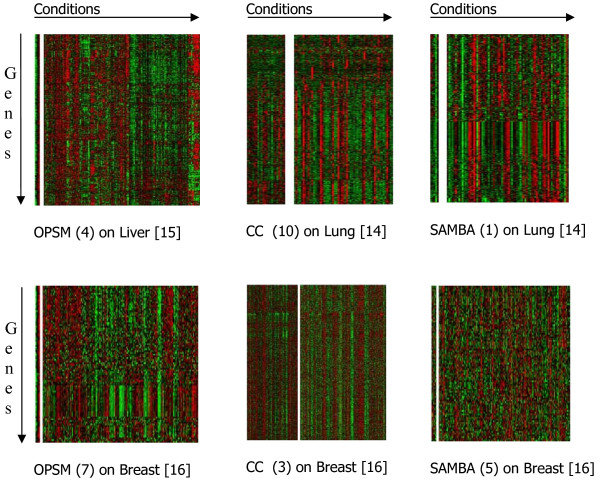
**Biclusters with comparable co-expression of the bicluster genes across non-bicluster conditions**. Heatmaps (red for induction and green for repression, genes are indicated in rows and conditions are shown in columns) of biclusters with comparable co-expression of the bicluster genes across non-bicluster conditions. In each figure, the left heatmap shows expression of the bicluster genes (rows) in the bicluster conditions (columns) and the right heatmap shows expression of the bicluster genes in the remaining conditions. All of them were chosen from top 10 biclusters output by the respective algorithms, the rank is indicated in the parenthesis.

Only limited efforts have been made to compare the performance of various biclustering algorithms on real data and nearly no effort has been made to combine the biclusters output by different biclustering algorithms into a single ranking. Ayadi et al [6] and Prelic et al [11] compared biclustering algorithms mainly using idealized simulated data which may not be reflective of the real data such as gene expression in tumors datasets. In addition, the focus was on evaluating the biclustering algorithms based on their ability to retrieve the idealized simulated biclusters i.e. co-expression is simulated only for genes in the bicluster in the conditions of the bicluster. It is a highly limited evaluation of biclustering algorithms as the real data is much more complex. If we have simulated an expression data of S conditions with one bicluster as follows: *X*_*ij *_= *N *(0, 1) with co-expression for *s *∈ *S *(all conditions) for *|s| *« *|S|*. The application of anyone of CC, ISA, OPSM and SAMBA algorithms can find this bicluster partly or fully as its genes are not co-expressed in the non bicluster conditions |*S-s*| » |*s*|. Whereas, application of same algorithms on lung [14], liver [15] and breast cancer [16] datasets resulted in biclusters (belonged to the top 10 biclusters output by each algorithm) with genes showing co-expression in non bicluster groups of conditions, see the Figure 2. This problem is not unique to any one algorithm but holds true for all biclustering algorithms as their scoring functions mainly depend on the bicluster conditions only. The presence of co-expression at comparable or better levels in the non-bicluster conditions show that the co-expression and biology of the bicluster genes is not limited to the conditions in the bicluster but it is a global effect. Therefore, evaluation on idealized simulated bicluster data may not be sufficient to reveal true effectiveness of a biclustering algorithm.

On real data, Prelic et al's [11] evaluation was based on the number of gene ontology (GO) terms enriched for the biclusters. It may not be a good measure for four reasons: (1) it solely depends on the genes in the biclusters and does not account for the conditions involved; (2) GO terms may be highly enriched even for normal clusters of genes which may not lack co-expression in any subset of the conditions; (3) it does not distinguish between good biclusters from traditional clusters; and, (4) it may be subjective owing to the hierarchical structure of the GO.

Hence, it is important to develop an objective scoring function that works well on real data to assess the quality or goodness of biclusters and hence the reliability of the biclustering algorithms. It will also be helpful in combining the results of applying different biclustering algorithms on a data into a single unified ranking, i.e. a meta-biclustering, which has not been addressed so far. It would be of great help as it facilitates best utilization of all biclustering algorithms as different algorithms may behave differently on different datasets.

In this paper we propose to develop such a scoring function based on differential co-expression framework similar to that proposed by Kostka and Spang [17]. In this framework, for a given bicluster, we fit two linear models for the expression of genes in the bicluster for the conditions in the bicluster and for the remaining (the non-bicluster) conditions separately. The resultant models are used together to assess goodness of the bicluster using our differential co-expression scoring function. Note that the aim of this paper is not to assess the efficiency of the biclustering algorithms in retrieving underlying biclusters in the data, but to assess how good the identified biclusters are and how to provide a good unified ranking of the biclusters (meta-biclustering algorithm) output by them. Using our scoring function we compare the performance of different biclustering algorithms on six real datasets.

## Results

### Differential co-expression framework for biclustering

Suppose we are given two microarray data matrices ( and ) related to a bicluster of I genes and J_1 _conditions: one is obtained from J_1 _bicluster conditions (aka group G_1_) and the other is obtained from J_2 _non-bicluster conditions (aka group G_2_); J_1_+J_2 _= M, the total number of conditions in the study. Each row corresponds to a gene and each column corresponds to a condition. Note that I is used to indicate both gene set and its cardinality, similar interpretation holds for the other sets of genes and conditions. The task is to find how well I genes form a bicluster on J_1 _conditions compared to the J_2 _conditions. If  is a good bicluster then there should be a co-expression of I in J_1 _and a clear differential co-expression of I between J_1 _and J_2 _conditions. To find it, we employ the framework developed for differential co-expression by Kostka and Spang [17], based on the linear modeling used by Cheng and Church [5], for both groups of conditions G_1 _and G_2_. Specifically, the linear model for the expression of I genes in the condition group G_k _is as follows:(1)

1 ≤ i ≤ I; 1 ≤ j ≤ J_k_; 1 ≤ k ≤ 2

Where *X*_*ijk *_is the log-expression of gene g_i _in condition p_jk _belonged to group G_k_. It is modeled as a summation of four factors: *μ*_k_, effect of group (overall effect) G_k_; *τ*_ik_, effect of gene *g*_*i *_in *G*_*k*_; *β*_*jk*_, effect of condition p_jk _in G_k_; and, *ε*_ijk_, an iid random error or residual of *g*_*i *_in p_jk_. Based on this model, Kostka and Spang's procedure obtains the mean of the squared residuals (*E*_*k*_) to score a set of genes I on J_k _conditions as follows:(2)(3)(4)

, ,  are the estimates of *τ*_ik_, *β*_jk_, and -*μ*_k _respectively.

**Figure 3:**
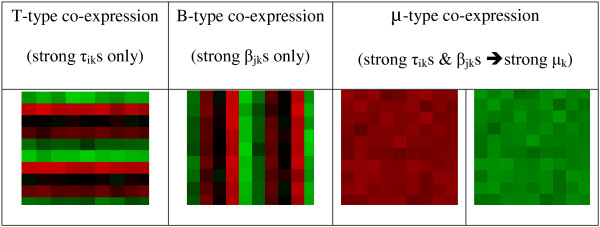
**Different types of co-expression**. Heatmaps (red for induction and green for repression, genes are indicated in rows and conditions are shown in columns) illustrating 3 types of co-expression: (1) T-type, gene effects only; (2) B-type, condition effects only; and, (3) *μ*-type, gene and condition effects.

The above linear modeling can elicit three different types of co-expression corresponding to different relative strengths of the parameters (*τ*_ik_, *β*_*jk *_and *μ*_*k*_) shown by four heatmaps in the Figure 3: (1) **T**-type co-expression; (2) **B**-type co-expression; and (3) **μ**-type co-expression. T-type co-expression is depicted by strong gene only effects resulting in strong *τ*_ik_s only as the effect of any condition over I is weak leading to weak or near-zero *β*_jk_s and *μ*_k_. B-type co-expression results from strong condition only effects leading to strong *β*_jk_s only as the overall expression of a gene across the bicluster conditions is weak leading to weak or near zero *τ*_ik_s and *μ*_k_. But, *μ*-type co-expression results due to the presence of strong gene as well as strong condition effects (strong *τ*_ik_s and *β*_jk_s) leading to strong *μ*_k_. We use the coefficients *τ*_ik_s, *β*_jk_s to quantify different types of co-expression, which is the first step to quantifying differential co-expression, of I genes in J_1 _and J_2 _conditions. T_k_(b) and B_k_(b) quantify the T-type and B-type co-expression of genes in a bicluster *b*:

for k = 1 and 2

I(b) is the number of genes in b and J_k_(b) is number of conditions in G_k _for b. Similar interpretation holds for the other variables also.

***Theorem***: T_k _and B_k _are the unbiased estimators of  and  respectively under the assumption that the noise in *X*_*ijk *_follows *N*(0, )

**Proof:**

As *E*_*k *_is an unbiased estimator of ,  is an unbiased estimator of *β*_*k*_. Similarly  is an unbiased estimator of *Γ*_*k*_.   ■

In the above proof,  is a non-central Chi-square distribution with 'n' degrees of freedom and 'c' being the non-centrality parameter; ⟨Z⟩ is the expectation of the random variable *Z*.

#### Scoring goodness of biclusters

The co-expression patterns in the biclusters output by any biclustering algorithm fits well into this categorization. A bicluster with no co-expression of any type for the bicluster genes in the non-bicluster conditions is the true bicluster. Comparable co-expression in the non-bicluster conditions means the conditions in the bicluster are not distinctive enough from the remaining conditions and hence do not qualify to be a bicluster. In such a case, the bicluster genes with all conditions in the study can be considered as a gene cluster with a strong co-expression across all conditions. Hence, biclustering fits well into differential co-expression framework. Then the differential co-expression score for bicluster b, SB(b) is

where 0<a<<1, it is a small fudge factor to offset large ratios based on very small co-expression in both groups of a bicluster. Strong positive SB(b) indicates strong co-expression in G_1 _and weaker or no co-expression in G_2 _vice versa.

Though we score a bicluster based on its differential co-expression, our quantification of differential co-expression by SB(b) is different from that used by Kostka and Spang, the S(b) = LOG(E_1_(b)/E_2_(b)), and their variance standardization approach for two reasons: (1) S(b) accounts mainly for B-type co-expression; and, (2) variance standardization does not account for different signal variances in the two groups.

#### Stratifying biclusters

After having selected significant biclusters based on SB(.), it is now important to stratify the biclusters into different types of co-expression. To achieve it, we define the following stratification score TS_k_(b) on the k^th ^group which is declared to be co-expressed by SB(b):

where k = 1 if SB(b) > 0

= 2 if SB(b) < 0

Large positive TS_b_(I) means the bicluster is of T-type (strong gene effects only), large negative score means the bicluster is of B-type (strong condition effects only) and small score close to 0 means they are of *μ*-type (strong gene as well as condition effects). Therefore, user can define a parameter *φ *> 0 to identify these three groups as follows:

### Evaluating Biclustering Algorithms and Combining Bicluster Lists

We have chosen four well-established biclustering algorithms for which software packages are available for evaluation and comparison (see Table 1 for summary): (1) CC, (2) ISA, (3) OPSM and (4) SAMBA. They are all aimed at identifying biclusters of genes co-expressed in a subset of conditions though they used different objective functions with a minor exception to OPSM which aims at identifying biclusters of order preserving co-expression. We used the respective default parameter settings for all these algorithms, similar evaluation may be carried out to combine the results obtained using different parameter settings. We have evaluated these biclustering algorithms on six real datasets from different biological domains: yeast to plant to different cancers. The summary of the datasets is given in Table 2. Each biclustering algorithm was applied on each data; CC, ISA and OPSM are applied using *BiCAT *toolbox [18] and SAMBA was applied using *EXPANDER *package [19]. The ranking of the biclusters by each algorithm is the ranking generated by the respective package. The biclusters with fewer than 5 conditions were filtered out from the evaluation as they appear to be strong because of the small number of conditions and may not be significant.

**Table 2:** Datasets used in the analysis

S. No	Dataset	Experiment	References	No. of Genes	No. of Samples
1	Breast	Breast Cancer	Wang et al. [16]	22283	286

2	Liver	Liver Cancer	Chen et al. [15]	10200	203

3	Yeast	Knock Out in Yeast	Gasch, et al. [20]	2993	173

4	Lymphoma	Lymphoma and Normal	Alizadeh, et al. [21]	4026	96

5	Lung	Lung Cancer	Broët et al. [14]	54837	79

6	Path_Metabolic	Plant	Wille et al. [22]	734	69

Datasets used in the analysis. The datasets are from diverse domains and of varying size.

We have evaluated the biclustering algorithms based on four criteria: (1) number of biclusters found; (2) median number of conditions in the biclusters; (3) ranking of the biclusters generated by an algorithm in the combined ranking of all biclusters generated by all algorithms; and, (4) types of biclusters generated.

**Figure 4:**
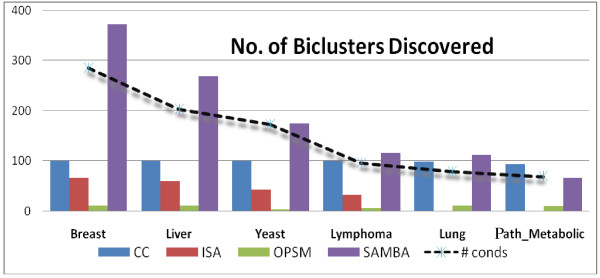
**Number of biclusters**. The number of biclusters (y-axis) output by different biclustering algorithms for 6 different datasets. The broken curve shows the number of conditions in each dataset.

The number of biclusters generated by different biclustering algorithms for each dataset is shown in the Figure 4. SAMBA has consistently output highest number of biclusters compared to any other algorithm. ISA has output moderate number of biclusters for large datasets (number of conditions > 100) and OPSM consistently output similar number (though small in number) of biclusters irrespective of the number of conditions. CC cannot be evaluated by this criterion as the number of biclusters is a parameter to the implementation of the algorithm. One striking pattern is that the performance in terms of number of biclusters output by both SAMBA and ISA does largely depend on the number of conditions in the dataset as shown by the trends, but OPSM is independent.

**Figure 5:**
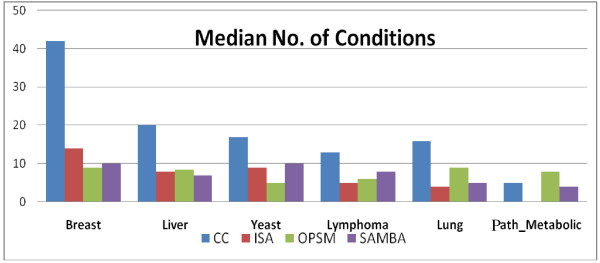
**Median number of conditions**. The median number of conditions (y-axis) in the biclusters output by different biclustering algorithms for 6 different datasets after filtering out small condition sized (<5) biclusters.

The histogram in the Figure 5 shows median number of conditions in the biclusters generated by each algorithm for different datasets. CC consistently output biclusters with very high number of conditions for all datasets except for *Path_Metabolic*. Median number of conditions used by CC strongly depends on the number of conditions in the dataset as seen by the trends; whereas ISA and SAMBA show a weak dependency on the same. Interestingly, OPSM does not show any dependency on the number of conditions in the dataset. Notably SAMBA, OPSM and ISA output biclusters of similar size.

**Figure 6:**
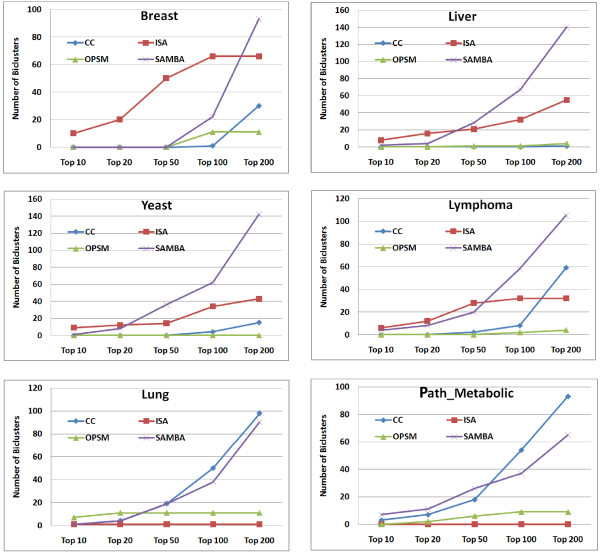
**Rank distribution of biclusters**. Rank distribution of the biclusters from each algorithm in a combined ranking on different datasets.

**Figure 7:**
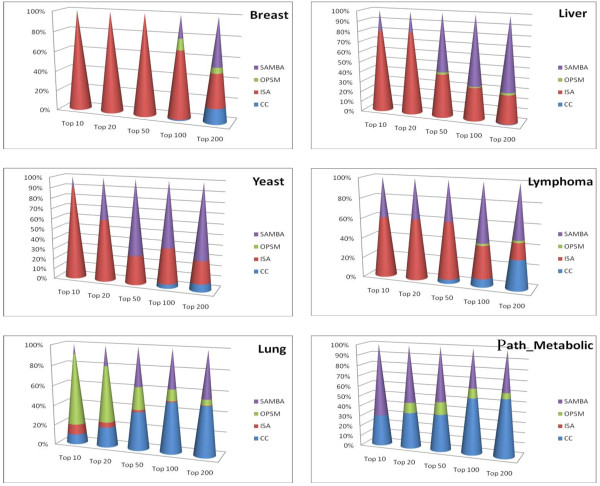
**Rank composition of top 100 biclusters**. Rank composition of the top 100 biclusters obtained by combined ranking of biclusters from each algorithm on 6 different datasets. The rank is shown on x-axis and the percent contribution of each algorithm is shown on y-axis.

Next, we turned to evaluating the goodness of the biclusters. For each dataset, we have combined the biclusters output by all algorithms into a single ranking based on our SB(b) score. Then we obtained the distribution of the biclusters output by each algorithm in this unified ranking as shown in the panels of plots in the Figures 6 and 7. For large datasets (Breast and Liver), the biclusters output by ISA appeared to be of higher goodness compared to the other biclustering algorithms. The goodness of the biclusters output by SAMBA is comparable to that of ISA for moderately large datasets (Yeast and Lymphoma) though it appears to be inferior to ISA for very large datasets (Breast). The goodness of the biclusters output by CC is consistently inferior to SAMBA and ISA on all medium and large datasets, it performs comparably only on small size datasets (Lung and Path_Metabolic) which appears to be consistent with the Prelic et al's results. OPSM does surprisingly better than the other algorithms only on Lung dataset and performs poorly on all other datasets. On the whole, it appears that the performance of SAMBA is consistently good across datasets of varying sizes. ISA appears to be good for large and very large datasets. CC and OPSM appear to be performing comparably on small datasets.

**Figure 8:**
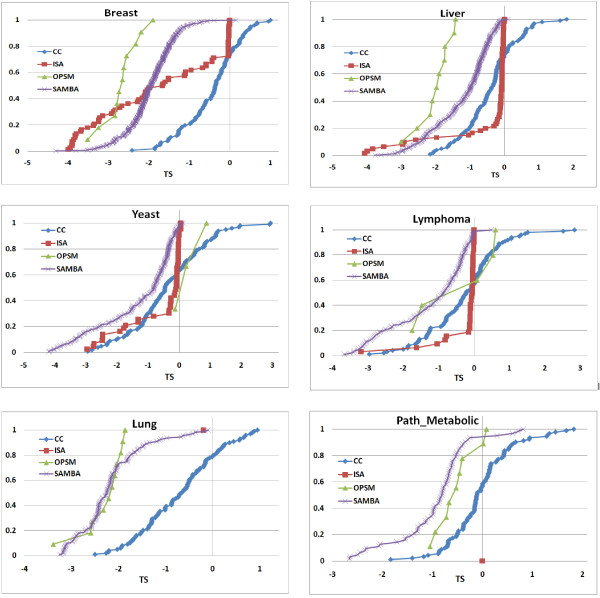
**Stratification of biclusters**. Cumulative distribution TS_1_(b) of the biclusters from each algorithm on 6 datasets. Highly negative TS_1_(b) (< -1) shows B-type co-expression, highly positive TS_1_(b) (> 1) shows T-type co-expression and TS_1_(b) close to zero (-1< TS_1_(b) <1) indicates *μ*-type co-expression.

**Figure 9:**
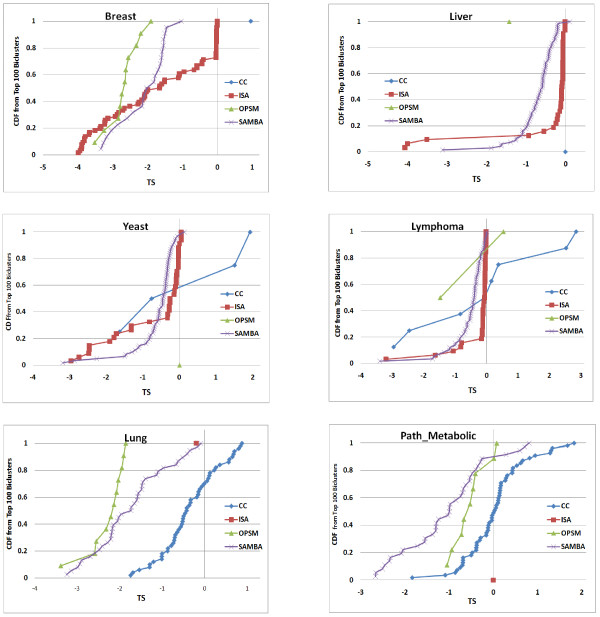
**Stratification of top 100 biclusters**. Cumulative distribution TS_1_(b) of the biclusters from each algorithm on 6 datasets contributing to the top100 biclusters from combined ranking. TS_1_(b) < -1 shows B-type co-expression, TS_1_(b) > 1 shows T-type co-expression and -1 < TS_1_(b) <1 indicates *μ*-type co-expression.

Further, we characterized the biclustering algorithms based on the types of co-expression found in their biclusters for all 6 datasets. It is assessed by using our bicluster stratification score TS_1_(b). We plot the cumulative distribution of the TS_1_(b) score of the biclusters output by each algorithm for each dataset as shown in the Figures 8 and 9, we set *φ *= 1. The behaviour of the algorithms does appear to be dependent on the dataset. ISA output ~60% of the biclusters of B-type for Breast, only 15%-20% for the other datasets. Apart from B-type, it output only *μ*-type biclusters and no T-type biclusters can be seen from ISA on any dataset. SAMBA output ~90% B-type in Breast and Lung, 40-50% in the remaining datasets. Strikingly, OPSM output only one type of biclusters for any dataset: only B-type biclusters were output on Breast, Liver and Lung datasets; only *μ*-type biclusters for Yeast, Lymphoma and Path_Metabolic datasets. This could be because OPSM identifies order preserving biclusters of B-type. Like ISA and SAMBA, OPSM also have not output any T-type biclusters on any dataset. Interestingly, only CC output biclusters of T-type and it output more of *μ*-type and T-type biclusters compared to B-type biclusters except on Breast data. On the whole it appears that all algorithms favoured B-type biclusters on Breast and Lung datasets and *μ*-type biclusters on Liver, Yeast and Lymphoma datasets.

## Discussion and Conclusions

Our study on real data has shown that evaluation of biclustering algorithms on idealized simulated data may not reflect the actual performance on real data owing to its complexity. So we proposed a conceptually and statistically sound framework based on the concept of differential co-expression to objectively compare the performance of the biclustering algorithms on real data and combine their output into a single unified ranking. This is based on the observation that a bicluster is revealed because the grouping of the bicluster genes could be strong only based on the bicluster conditions. As several biclustering algorithms do not consider the effect of non-bicluster conditions in the scoring and discovery of the biclusters, we found several biclusters with a strong grouping of genes based on the non-bicluster conditions also. This does not qualify them to be biclusters as the genes could be grouped nearly strongly even with all conditions together i.e. co-expression is more of a global effect. The strength of grouping can be represented by condition and gene effects and their differential between bicluster and non-bicluster conditions for the bicluster genes indicate true biclusters. We considered three types of co-expression unlike in a typical differential co-expression study and the ranking is based on the model coefficients rather than the model errors to reflect different types of co-expression. In this formulation, we explicitly estimate the effects of genes, conditions in bicluster conditions and non bicluster conditions. Strong effects of either genes or conditions would indicate co-expression of genes in the given group of conditions. Taking ratio of the co-expression scores between bicluster and non bicluster conditions gives us the measure of the goodness of the biclusters. Further we proposed a bicluster stratification score to classify the biclusters based on their co-expression patterns: high score means genes are co-expressed similarly across conditions in the bicluster, but the genes could be divided into two groups one with induction and the other with repression; low score means genes are co-expressed across conditions, conditions can be divided into two groups - one with induction of all genes and the other with repression; medium or near-zero score means all genes are either induced or repressed but not a combination in all conditions. The framework we used is analogous to ANOVA with T_k_, B_k _and *μ*_k _being similar to the variance terms with null centrality parameter being '0'.

We have compared four well known biclustering algorithms: ISA, OPSM, CC and SAMBA. Their application on six different datasets revealed that ISA outputs the best biclusters but its performance is dependent on the number of conditions in the dataset; SAMBA performs well on all datasets of the varying number of conditions; though OPSM does not perform well on most datasets, it is still useful on certain datasets like Lung cancer data; whereas CC outputs least goodness biclusters with high stratification scores. Further, there is a data dependency on the types of co-expression present in the biclusters: all algorithms output predominantly B-type biclusters on Breast and Lung datasets and a mix of B-type and *μ*-type biclusters for Liver, Yeast and Lymphoma datasets, though *μ*-type biclusters are slightly more in number. Strikingly, OPSM output mostly B-type biclusters and CC is the only algorithm output T-type biclusters.

However, the evaluation presented in the paper may vary with a change in parameter settings of the individual algorithms. But it is helpful even to compare different parameter settings for a given algorithm and choose suitable parameter settings. Hence, the scoring function is useful, as shown here, to get unified ranking of the biclusters (i.e. meta-biclustering algorithm) produced by different algorithms for different parameter settings. However, we are working on devising an algorithm based on the differential co-expression framework as it may find novel biclusters with strong differential co-expression.

Moreover, though the proposed goodness scoring function is tailored to assess the goodness of the biclusters of co-expressed genes, the general framework of differential co-expression can be extended to evaluate the goodness of the other types of biclusters such as low error in the expression which requires a scoring function proposed by Kostka & Spang i.e. ratio of error variances = E_2_(b)/E_1_(b).

## Competing interests

The authors declare that they have no competing interests.

## Authors' contributions

CKBH conducted all experiments and participated in the development of the work. RKMK developed and led the project, also written the paper. All authors read and approved the final manuscript.
